# Deprotonation of retinal Schiff base and structural dynamics in the early photoreaction of primate blue cone visual pigment

**DOI:** 10.1016/j.bpj.2025.05.004

**Published:** 2025-05-07

**Authors:** Yosuke Mizuno, Kota Katayama, Hiroo Imai, Hideki Kandori

**Affiliations:** 1 Department of Life Science and Applied Chemistry, Nagoya Institute of Technology, Showa-ku, Nagoya, Japan; 2 OptoBioTechnology Research Center, Nagoya Institute of Technology, Showa-ku, Nagoya, Japan; 3 Center for the Evolutionary Origins of Human Behavior, Kyoto University, Inuyama, Japan

## Abstract

Animal rhodopsin is a photoreceptive protein crucial for vision, with activation triggered by the *cis-trans* isomerization of a retinal chromophore upon light absorption. This activation involves a series of thermal intermediates, ultimately leading to G protein-mediated signal transduction. The retinal chromophore is covalently bound to the protein through a protonated Schiff base, and its deprotonation during the formation of the active intermediate is believed to induce structural changes in α-helices that facilitate G-protein interactions. Using low-temperature UV-visible absorption and Fourier transform infrared spectroscopy, we investigated the early photoreaction of the primate blue cone visual pigment (MB). Our results demonstrate that Schiff base deprotonation in the early photoreaction is coupled with local perturbations in α-helices, promoting the formation of the Lumi intermediate. Using site-directed mutagenesis, we identified the proton acceptor involved in Schiff base deprotonation and mapped the regions of α-helical structural changes during the formation of the Lumi intermediate. We discovered that the proton released from the Schiff base is transferred to the counterion Glu113. Systematic mutagenesis revealed that structural perturbations in transmembrane helix 7 bring Glu113 and the lysine residue forming the Schiff base into proximity, facilitating efficient proton transfer during the early photoreaction. Additionally, the Lumi intermediate formed at low temperatures was found to revert to the original state through thermally driven reverse proton transfer, coupled with retinal reisomerization. From an evolutionary perspective, MB is part of a group of UV-sensitive cone visual pigments characterized by a deprotonated retinal Schiff base in the ground state. The observed propensity for MB to undergo Schiff base deprotonation is consistent with this evolutionary trait.

## Significance

Understanding early photoreaction mechanisms of animal rhodopsins is fundamental to elucidating vision at the molecular level. Here, we reveal that Schiff base deprotonation in the primate blue cone visual pigment (MB) is coupled with the local structural perturbations in α-helices, promoting the formation of the Lumi intermediate. By identifying the proton acceptor and mapping α-helical changes, we provide insights into the role of transmembrane helix 7 in facilitating proton transfer. These findings enhance our comprehension of visual pigment activation and support an evolutionary link between MB and UV-sensitive cone pigments with a deprotonated retinal Schiff base in the initial state.

## Introduction

The primate visual system, including that of humans, relies on two distinct types of photoreceptive molecules to process light across a range of environmental conditions: rod photoreceptor pigment (rhodopsin), which is highly sensitive and enables vision in low-light environments by detecting light and dark contrasts, and cone photoreceptor pigment (cone opsin), which functions under bright light and supports color vision by being sensitive to specific wavelengths (1,2,3). Human color vision is fundamentally based on the perception of the three primary colors of light (RGB), a phenomenon directly linked to the presence of three distinct cone pigments, each attuned to red, green, or blue light (3,4,5).

Both rhodopsin and cone pigments share a conserved molecular architecture, consisting of a protein component, opsin, and a chromophore, 11-*cis*-retinal, which together mediate light absorption. The spectral sensitivity of each pigment is primarily determined by structural variations in the opsin proteins (3,6). Despite their shared structural foundation, rods and cones exhibit markedly different functional properties. Rods are distinguished by their extremely high sensitivity to light, enabling vision in dimly lit environments. In contrast, cones are characterized by faster photoresponse kinetics, rapid adaptation to bright light, and a crucial role in high-acuity vision. The distinct physiological and molecular differences are governed by the reaction dynamics of the pigments (4).

The mechanisms of light absorption and signal transduction in rhodopsin have been extensively studied and elucidated at the atomic and molecular levels (3). These advancements have been made possible through the integration of various spectroscopic, biochemical, and structural techniques, including x-ray crystallography (7,8,9,10,11,12,13,14), electron microscopy (15,16,17,18), and infrared spectroscopy (19,20,21,22,23,24). Collectively, these studies have provided a thorough understanding of rhodopsin’s essential role in coordinating the visual response, wherein photonic signals are converted into electrical impulses and transmitted from rod cells to the brain via the optic nerve.

Upon light absorption, rhodopsin undergoes a series of well-defined structural transitions driven by thermal relaxation. These transitions include the formation of photorhodopsin, bathorhodopsin (Batho), lumirhodopsin (Lumi), and metarhodopsin-I (Meta-I), ultimately leading to the formation of metarhodopsin-II (Meta-II), the active intermediate responsible for G-protein activation. G-protein activation is followed by hydrolysis of the retinal Schiff base linkage. Notably, in Meta-II, the proton from the retinal Schiff base is transferred to Glu113, its counterion, triggering a large-scale conformational change in the receptor. This structural rearrangement significantly enhances G-protein activation efficiency, enabling rhodopsin to achieve its characteristic high sensitivity to light (3).

In contrast, invertebrate rhodopsins and nonvisual opsins (e.g., Opn3, Opn4, and Opn5) often retain a protonated Schiff base even in their active metarhodopsin states (25). This persistent protonation is believed to limit large-scale structural rearrangements, leading to a relatively lower G-protein activation efficiency compared with vertebrate rhodopsins (3).

These observations emphasize the crucial role of proton transfer reactions in the light-driven activation of rhodopsin, highlighting their essential contribution to the receptor’s functional performance and its ability to mediate visual sensitivity.

Cone pigments are thought to undergo a photoactivation process similar to rhodopsin, as they share a common structural motif of seven transmembrane helices and use 11-*cis*-retinal as the chromophore (4). However, significant differences have been observed among these pigments in terms of the lifetime of their photoactivated state, photosensitivity, and regeneration kinetics. Previous temperature-dependent UV-visible spectroscopic studies of chicken cone pigments showed that retinal Schiff base deprotonation occurs exclusively during the formation of the Meta-II intermediate, with all preceding intermediates remaining protonated (26).

More recently, ultrafast time-resolved transient absorption spectroscopy studies have been conducted on human green (HG) (27) and human blue (HB) cone pigments (28). These studies revealed that, while the kinetics of Meta-II formation are similar between bovine rhodopsin and HB, the Meta-I to Meta-II transition in HB occurs nearly an order of magnitude faster than in HG. However, the isolation and preparation of cone pigments remain technically challenging, limiting the progress of structural studies intended to uncover the underlying differences in their physicochemical properties.

To date, we have conducted structural studies on primate cone pigments recombinantly expressed in mammalian systems using light-induced difference Fourier transform infrared (FTIR) spectroscopy. Light-induced difference spectroscopic studies comparing the Batho and initial states at 77 K revealed that primate blue (MB), green (MG), and red (MR) pigments, along with rhodopsin, share a common retinal structure and *cis*-*trans* isomerization, albeit with slightly different torsion patterns along the polyene chain (29,30). Notably, for MB, the formation of the BL intermediate after the Batho intermediate was observed at 163 K (31). FTIR spectral analysis showed that the distortion in the retinal chromophore caused by the formation of the Batho intermediate is relaxed in the BL intermediate, which is an intermediate formed between Batho and Lumi, as assigned in chicken cone pigments (26). The exclusive observation of the BL intermediate in MB is consistent with recent ultrafast time-resolved transient absorption spectroscopy studies on HB (28).

Additionally, FTIR spectra of the Lumi intermediate were successfully obtained for MG at 200 K (32) and for MB at 223 K (33). In MG, the intermediate was formed by transition from the Batho state, and in MB, it was formed by transition from the BL state. Therefore, both intermediates were assigned to Lumi (32,33). Notably, the infrared signals associated with retinal distortional relaxation and significant structural changes in the α-helices during Lumi intermediate formation were found to be similar between MG and rhodopsin. In contrast, the Lumi intermediate of MB exhibited retinal Schiff base deprotonation, accompanied by more extensive structural changes in the α-helices compared with MG and rhodopsin, as indicated by distinct infrared signals.

To date, there have been no reports of retinal Schiff base deprotonation occurring during the early stages of photoreaction in visual pigments. This raises several unresolved questions, such as the identity of the proton acceptor, the structural basis that facilitates proton transfer, and the distinct mechanism responsible for the early proton transfer reaction observed exclusively in MB.

In this study, we employed a series of systematically designed mutants of MB to investigate the structural basis facilitating proton transfer reactions in the Lumi intermediate and to identify the proton acceptor from the retinal Schiff base. Additionally, we aimed to elucidate why proton transfer reactions are uniquely observed during the early photoreaction processes of MB. Our findings reveal that Glu113, the counterion, serves as the primary proton acceptor from the retinal Schiff base.

To further investigate the molecular mechanisms underlying these phenomena, we focused on the multiple cysteine residues present in MB. Systematic cysteine mutants were generated, and FTIR spectroscopy was used to examine the S-H stretching vibrations of these residues. This analysis revealed that the significant structural changes in the α-helix, which occur specifically during the formation of the Lumi intermediate, are localized in the transmembrane helix 7 (TM7) region, particularly near the Schiff base. These structural changes reduce the distance between the Schiff base and Glu113, facilitating proton transfer reactions during the Lumi intermediate stage.

Furthermore, we found that the Lumi intermediate reverts to the initial state through thermal retinal reisomerization, followed by a subsequent proton return reaction. These results offer a mechanistic explanation for the observation that proton transfer reactions are exclusive to MB. Our findings provide valuable insights into the early photoreaction dynamics and the molecular architecture of blue cone pigments, enhancing our understanding of its unique functional properties.

## Materials and methods

### Sample preparation

The cDNA encoding the primate blue pigment (UniProt ID: F7GQA6, short-wave-sensitive opsin 1 from the white-tufted-ear marmoset) was tagged with the Rho1D4 epitope sequence and inserted into the pFastBac HT expression vector (Bac-to-Bac, Thermo Fisher Scientific) using the in-fusion cloning method. Site-directed mutagenesis was carried out using the QuikChange Site-Directed Mutagenesis Kit (Agilent Technologies, Santa Clara, CA). The resulting constructs were expressed in *Spodoptera frugiperda* (Sf9) cells cultured in ESF921 medium (Expression Systems) at 28°C. After 48 h of incubation, the infected cells were harvested by centrifugation and stored at −80°C. Cell pellets were resuspended in a low-salt buffer (25 mM HEPES, 20 mM KCl, 10 mM MgCl_2_ [pH 7.5]) containing a protease inhibitor (Sigma-Aldrich, Japan) using a Dounce homogenizer. The suspension was centrifuged for 20 min, and the process was repeated at least twice. Subsequently, the pellets were resuspended in a high salt buffer (25 mM HEPES, 20 mM KCl, 10 mM MgCl_2_, 1 M NaCl [pH 7.5]) supplemented with a protease inhibitor (Sigma-Aldrich) and benzonase nuclease (Novagen) to improve solubilization efficiency and remove nucleotides. The resulting pellets were then resuspended in Pm buffer (50 mM HEPES, 140 mM NaCl [pH 7.0]) at a ratio of 25 mL of buffer per 1 L pellet of insect cell culture, and regenerated with 30 *μ*M of 11-*cis*-retinal by overnight stirring at 4°C. The regenerated sample was solubilized in solubilization buffer (1% [w/v] *n*-dodecyl-β-D-maltoside [DDM], 50 mM HEPES, and 140 mM NaCl [pH 7.0]) and centrifuged at >147,600 × *g* for 40 min using a fixed-angle rotor. The supernatant was incubated with 1D4 antibody-conjugated resin for 4 h. The resin was washed with wash buffer (50 mM HEPES, 140 mM NaCl, and 0.02% DDM [pH 7.0]), and the bound sample was eluted with elution buffer (0.40 mg/mL 1D4 peptide, 50 mM HEPES, 140 mM NaCl, 0.02% DDM [pH 7.0]).

### Sample preparation for spectroscopic measurements

For spectroscopic measurements, the purified sample was reconstituted into phosphatidylcholine (PC) liposomes. The protein was mixed with a reconstitution buffer containing 50 mM HEPES, 140 mM NaCl, 0.75% CHAPS, and 1 mg/mL PC at a protein/lipid molar ratio of 1:30. Detergent removal was achieved by dialysis, and the reconstituted sample was suspended in a buffer containing 2 mM NaH_2_PO_4_ (pH 7.25) and 5 mM NaCl. The reconstituted sample was deposited onto a BaF_2_ window to form dry films, and then hydrated films were prepared by dropping the 1 *μ*L of H_2_O or D_2_O beside dry films for the subsequent spectroscopic measurements.

### Low-temperature UV-visible and FTIR spectroscopies

Hydrated films were mounted on a cryostat (Optistat, Oxford Instruments) attached to a UV-visible spectrophotometer (V750, JASCO) and an FTIR spectrometer (Cary670, Agilent).

In low-temperature UV-visible spectroscopy, intermediates were generated by irradiating the sample with specific wavelengths using interference filters: 420 nm for 5 min, 400 nm for 3 min, 380 nm for 5 min, or >430 nm using a cutoff filter for 5 min. Difference spectra were calculated by subtracting the spectrum recorded before light irradiation from the spectrum obtained after irradiation.

For FTIR spectroscopy, the BL intermediate was accumulated at 163 K by irradiating with a 400 nm interference filter for 5 min and reverted to the dark state by irradiating with >480 nm using a cutoff filter for 5 min. This process was conducted for WT, E113D, T118C, C87S, C211S, C262S, C264S, and C299S variants. At 223 K, the BL intermediate was generated by irradiating with a 380 nm interference filter for 5 min in WT or a 400 nm interference filter for 3 min in E113D. Reversion to the dark state was achieved by irradiation with >500 nm (cutoff filter) for 5 min in WT or >450 nm (cutoff filter) for 3 min in E113D. The Lumi intermediate for all mutants was generated by irradiation with >430 nm (cutoff filter) for 5 min and reverted to the dark state by incubation in the dark for 10 min. Difference spectra were calculated by subtracting the spectrum before irradiation from the spectrum after irradiation.

For thermal reisomerization experiments using UV-visible spectroscopy, the Lumi intermediate was accumulated by irradiation with >430 nm (cutoff filter) for 10 min at 213, 223, or 233 K, followed by incubation in the dark. Spectra were recorded every 2 min over 3 h, and each experiment was repeated three times at each temperature. Time-course data were generated by plotting the intensity difference between 344 and 421 nm. These plots were fitted to a double-exponential function, and time constants were calculated. Activation energies were estimated using an Arrhenius plot fitted with a linear function.

### pH titration

pH titration was performed using solubilized samples purified with 1D4 antibody. Samples were adjusted to an optical density of 0.35 at 415 nm and diluted in a mixed buffer solution (20 mM citrate, 20 mM HEPES, 20 mM MES, 20 mM MOPS, 20 mM CHES, 20 mM CAPS, 100 mM NaCl, 0.02% [w/v] DDM [pH 7.0]). The pH was increased using NaOH, and spectra were recorded at intervals of 0.25 pH units.

## Results

### Glu113 serves as the proton acceptor from the retinal Schiff base

**Figure 1:**
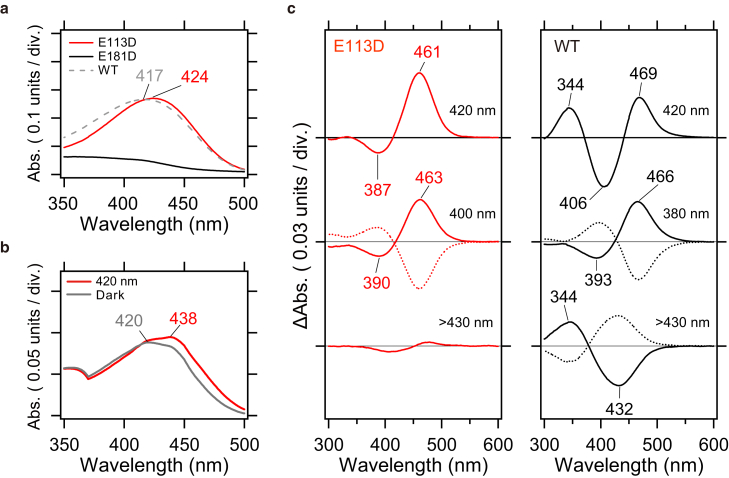
Identification of proton acceptor using UV-visible absorption spectroscopy. (*a*) Comparison of UV-visible absorption spectra of purified samples: E113D (*red line*), E181D (*black line*), and WT (*gray dashed line*). (*b*) UV-visible spectra of E113D at 223 K in the dark (*gray line*) and after illumination at 420 nm (*red line*). (*c*) Comparison of UV-visible difference spectra before and after illumination for E113D and WT at 223 K. Left panel (E113D). Top: difference spectra before and after illumination at 420 nm. Middle: difference spectra before and after illumination at 400 nm (*solid red line*) and >450 nm (*dashed red line*). Bottom: difference spectra before and after illumination at >430 nm. Right panel (WT). Top: difference spectra before and after illumination at 420 nm. Middle: difference spectra before and after illumination at 380 nm (*solid black line*) and >500 nm (*dashed black line*). Bottom: difference spectra before and after illumination at >430 nm (*solid black line*) and 360 nm (*dashed black line*).

To identify the proton acceptor from the retinal Schiff base during the formation of the MB Lumi intermediate, site-directed mutants were created targeting the carboxylic acids Glu113 and Glu181, which are potential candidates near the retinal Schiff base. Fig. 1 *a* compares the UV-visible absorption spectra of purified samples of the E113D, E181D, and wild-type (WT) variants. The WT exhibits a maximum absorption wavelength (λ_max_) at 417 nm, while the E113D mutant shows a 7 nm red shift to 424 nm. Generally, a stronger interaction between the protonated retinal Schiff base and its counterion results in a blue shift of λ_max_, whereas a weaker interaction leads to a red shift (34). In this case, the red shift observed in E113D is likely due to the shortening of the carboxylic acid side chain by one carbon, providing strong evidence that Glu113 serves as the counterion.

In contrast, as shown in Fig. 1 *a*, the E181D mutant did not exhibit any visible absorption. Fig. S1 presents the results of mutating Glu181 to Asp, Gln, and Ala, respectively, but none of these mutants displayed absorption. Western blot analysis confirmed the successful expression of all three mutants, suggesting that the mutation at Glu181 disrupts retinal binding.

To further confirm whether Glu113 is the proton acceptor from the Schiff base, the photoreaction of the E113D mutant at low temperatures was examined. When the WT is illuminated with light at 420 nm at 223 K, both the red-shifted BL intermediate (469 nm) and the blue-shifted Lumi intermediate (344 nm), corresponding to Schiff base deprotonation, are observed (Fig. 1 *c*, *right*/*top panel*) (33). This suggests that the BL and Lumi intermediates are in equilibrium at 223 K. Additionally, this equilibrium can be shifted toward the BL intermediate with 380 nm light illumination (Fig. 1 *c*, *right*/*middle panel*) and the Lumi intermediate with >430 nm light illumination (Fig. 1 *c*, *right*/*bottom panel*). As shown by the dotted lines in Fig. 1 *c* (*right*/*middle* and *right*/*bottom panels*), both BL and Lumi intermediates exhibit photoreversible properties (photochromism).

In the case of E113D, illumination with 420 nm light at 223 K caused a red shift in the λ_max_ (Fig. 1 *b*), and the difference spectrum revealed a single peak at 461 nm in the visible region (top spectrum in the *left panel* of Fig. 1 *c*). This red-shifted intermediate exhibited photochromism similar to the BL intermediate of WT. Upon 400 nm illumination, the difference spectrum showed a negative peak at 390 nm and a positive peak at 463 nm (Fig. 1 *c*, *left*/*middle panel*), closely resembling the spectral features of the WT BL intermediate (393 nm (−)/466 nm (+)). However, no absorption was observed, including in the UV region, when E113D was illuminated with >430 nm light. These results strongly suggest that Schiff base deprotonation does not occur in E113D, providing compelling evidence that Glu113 is the proton acceptor from the Schiff base during the formation of the Lumi intermediate in WT.

### Comparison of light-induced FTIR difference spectra of MB WT and E113D

**Figure 2:**
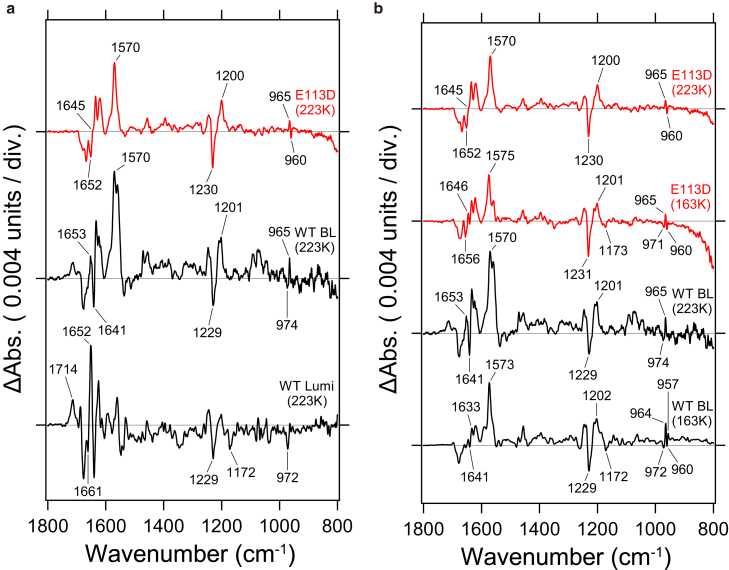
FTIR spectral characteristics of the E113D mutant. (*a*) Comparison of FTIR difference spectra of E113D and WT before and after light illumination at 223 K measured in H_2_O hydration. Top: difference spectra of E113D. Middle: difference spectra between BL intermediate and initial state in WT. Bottom: difference spectra between Lumi intermediate and initial state in WT. (*b*) FTIR difference spectra of E113D and WT at 223 and 163 K measured in H_2_O hydration. Top: difference spectra of E113D at 223 K. Second: difference spectra of E113D at 163 K. Third: difference spectra between BL intermediate and initial state in WT at 223 K. Bottom: difference spectra between BL intermediate and initial state in WT at 163 K. One division of the *y* axis corresponds to 0.004 absorbance units.

In E113D, it was observed that the proton transfer reaction from the Schiff base does not occur at 223 K, resulting in the accumulation of the BL intermediate. To investigate potential structural differences between the BL intermediate of E113D and that of WT, low-temperature FTIR spectroscopy was performed. Fig. 2 presents the light-induced FTIR difference spectra of E113D measured at 223 and 163 K. As shown in Fig. 1, WT forms both the BL and Lumi intermediates at 223 K, with their respective spectra depicted in the middle and bottom of Fig. 2 *a* (33). In WT, the negative band at 1229 cm^−1^ corresponds to the C-C stretching vibration of 11-*cis*-retinal in the initial state, identified as the C_12_-C_13_ stretching vibration based on previous studies of chicken red-sensitive visual pigments (35). In the BL intermediate, a positive band at 1201 cm^−1^, arising from the C-C stretching vibration of all-*trans*-retinal, is observed. In contrast, in the Lumi intermediate, where the Schiff base is deprotonated, the retinal C-C stretching vibration becomes infrared inactive, resulting in the absence of positive bands.

In E113D, measured at 223 K, a paired band at 1230 (−)/1200 (+) cm^−1^ was observed, corresponding to 11-*cis* and all-*trans*-retinal, respectively, indicating that the Schiff base was not deprotonated. Additionally, the positive 1714 cm^−1^ band, which originates from the protonated carboxylic acid observed in the WT Lumi intermediate, was absent in E113D. Furthermore, the amide-I band (1661 (−)/1652 (+) cm^−1^), which signifies significant structural changes in the α-helix due to the proton transfer reaction, was also not observed in E113D. These findings further support the conclusion that the proton transfer reaction did not occur.

Fig. 2*b* compares the spectra of E113D and WT at 223 and 163 K. In the WT BL intermediate measured at different temperatures (*black line* in Fig. 2 *b*), the retinal chromophore bands below 1300 cm^−1^ are nearly identical, suggesting no structural or electronic differences in the retinal chromophore. However, differences in the protein backbone were observed in the 1700–1500 cm^−1^ region. Specifically, in the WT BL intermediate at 163 K, an amide-I pair band appeared at 1641 (−)/1633 (+) cm^−1^, whereas at 223 K the shift direction reversed, and the amide-I pair band was observed at 1653 (+)/1641 (−) cm^−1^ with increased signal intensity. This change is attributed to local structural relaxation as the temperature increases.

In E113D, the spectra of the BL intermediate at 223 and 163 K were nearly identical across the 1800–800 cm^−1^ region. In the 1700–1500 cm^−1^ range, the 1656 (−)/1646 (+) cm^−1^ pair band at 163 K corresponds to the 1652 (−)/1645 (+) cm^−1^ pair band at 223 K. Unlike WT, E113D exhibited little to no temperature-dependent structural relaxation. In WT, this structural relaxation with increasing temperature is likely a precursor to the next step: the proton transfer reaction in the Lumi intermediate.

### Protein structural changes to form Lumi intermediate

In the WT Lumi intermediate spectrum, the proton transfer reaction from the Schiff base was accompanied by significant conformational changes in the α-helix, along with alterations in the hydrogen bonding of several amino acids. To further investigate these structural changes, we attempted to assign the relevant bands by analyzing amino acid mutations. Specifically, during the formation of the Lumi intermediate, structural changes leading up to deprotonation likely bring the Schiff base and its counterion, Glu113, into closer proximity, rather than this being a consequence of the deprotonation itself. This suggests that a localized movement of TM3, where Glu113 resides, likely occurs during this process.

**Figure 3:**
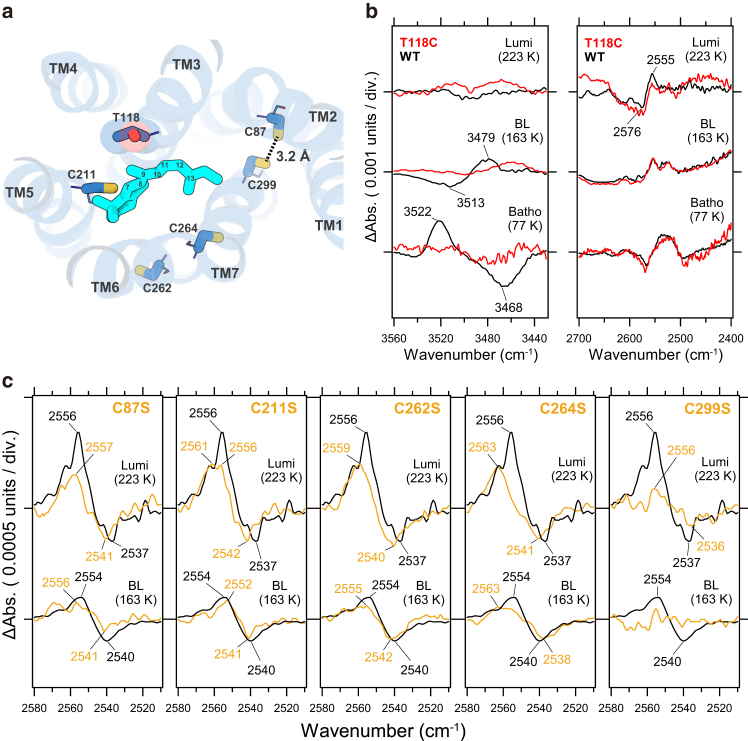
Spectral comparison of mutant proteins in the amino acid side chain vibrational mode region. (*a*) Structure of MB modeled using AlphaFold2, shown from a perspective viewed from the extracellular region. The side chains of amino acids subjected to site-directed mutagenesis are represented as sticks, while the main chain is depicted as a line representation. A hydrogen bond between Cys87 and Cys299, which are within hydrogen-binding distance, is indicated by the black dashed line. (*b*) Comparison of FTIR difference spectra of the T118C mutant (*red line*) and WT (*black line*) measured in D_2_O. Left (3560–3440 cm^−1^ region) and right panel (2700–2400 cm^−1^ region). Top: difference spectra between Lumi intermediate and initial state, Middle: difference spectra between BL intermediate and initial state. Bottom: difference spectra between Batho intermediate and initial state. One division of the *y* axis corresponds to 0.001 absorbance unit. (*c*) Comparison of FTIR difference spectra of cysteine mutants (*orange lines*: C87S, C211S, C262S, C264S, and C299S) and WT (*black line*) measured in H_2_O. Top: difference spectra between Lumi intermediate and initial state. Bottom: difference spectra between BL intermediate and initial state. The obtained spectra of WT, C87S, C211S, C262S, C264S, and C299S are scaled by 1, 2.4, 1.2, 1.6, 0.7, and 2.3, respectively.

In the spectra of the Batho and BL intermediates in the WT (*black line*) shown on the left side of Fig. 3 *b*, pair bands at 3522 (+)/3468(−) cm^−1^ and 3513 (−)/3479 (+) cm^−1^, were observed even under D_2_O hydration. Previous FTIR studies have shown that similar O-H stretch bands of Thr118 appear in bovine rhodopsin even in D_2_O hydration (36), indicating these bands likely originate from Thr118 in TM3, located near the C_9_ methyl group of the retinal (Fig. 3 *a*) (37). In the Lumi intermediate, these bands disappear. Two possible explanations for this disappearance were considered: first, that the hydrogen bonding environment of Thr118 reverted to its initial state upon formation of the Lumi intermediate, and, second, that the O-H stretching vibration of Thr118 shifted due to deuterium exchange, induced by the substantial structural changes in the α-helix specific to the Lumi intermediate. The 2576 (−)/2555 (+) cm^−1^ pair band observed on the right side of Fig. 3 *b* was proposed as a candidate for the shifted band.

To examine which of the two possibilities is correct, we analyzed the T118C mutant (*red line*) measured in D_2_O hydration. As expected, the pair bands observed in the Batho and BL intermediates disappeared on the left side of Fig. 3 *b*, confirming that these bands correspond to the O-H stretching vibrations of Thr118. However, the 2576 (−)/2555 (+) cm^−1^ pair band seen in the Lumi intermediate remained in the mutant on the right side of Fig. 3 *b*, suggesting that Thr118 was not deuterated during Lumi intermediate formation. This implies that the hydrogen bonding environment altered in the Batho and BL intermediates was restored. The origin of this pair band (2576 (−)/2555 (+) cm^−1^) will be further explored in future studies using a series of mutations. These findings indicate that, at least locally, TM3 adopts a structural state during Lumi intermediate formation that is similar to its initial conformation. This suggests that Glu113 also remains in a position that has not changed from the initial state. This raises the question: Is the distance between the Schiff base and its counterion truly decreasing?

Next, we focused on the S-H stretching vibration band of cysteine, a significant functional group change observed in the Lumi intermediate spectrum. As shown in Fig. 3 *c*, the pair bands at 2554 (+)/2540 (−) cm^−1^ in the BL intermediate and 2556 (+)/2537 (−) cm^−1^ in the Lumi intermediate under H_2_O hydration are attributed to the S-H stretching vibrations of cysteine, based on their frequencies. Additionally, deconvolution analysis using Gaussian fitting on each band is presented in Fig. S2. As seen in Fig. S2, two distinct S-H stretching vibration bands corresponding to cysteine were identified. In the BL intermediate, bands at 2567 (+), 2554 (+), and 2540 (−) cm^−1^ were detected, with the negative 2540 cm^−1^ band showing a broad profile, indicating a possible overlap of the S-H stretching vibrations from two cysteine residues. In contrast, for the Lumi intermediate, the analysis revealed bands at 2564 (+), 2555 (+), 2545 (−), and 2538 (−) cm^−1^.

MB contains 13 cysteine residues, including Cys87 in TM2, Cys211 in TM5, Cys262 and Cys264 in TM6, and Cys299 in TM7, all located near the retinal chromophore (Fig. 3 *a*). To assign the observed bands, we performed systematic measurements on mutants targeting the five cysteine residues closest to the retinal chromophore. As shown in Fig. S3, we successfully purified sufficient quantities of samples for FTIR measurements for all five cysteine mutants.

In the C87S mutant, the pair bands at 2554 (+)/2540 (−) cm^−1^ observed in the BL intermediate showed a slight reduction in intensity. In the Lumi intermediate, the positive band at 2556 cm^−1^ was significantly diminished, and the negative band at 2537 cm^−1^ was upshifted by 5 cm^−1^ to 2541 cm^−1^. In the C211S mutant, no effects were seen in the BL intermediate; however, in the Lumi intermediate, a notable reduction in the positive band at 2556 cm^−1^ and an upshift of the negative band were observed. Similarly, in the C262S and C264S mutants, the positive band at 2554 cm^−1^ in the BL intermediate was reduced by approximately half, and in the Lumi intermediate, a substantial decrease in the 2556 cm^−1^ band and an upshift in the negative band were noted. In contrast, the C299S mutant exhibited almost no S-H stretching vibration bands of cysteine in either the BL or Lumi intermediates. Fig. S4 presents a graph comparing the areas of the negative and positive bands for the BL and Lumi intermediates across the WT and various mutants. As shown, the most significant effect was observed in the C299S mutant, followed by the C87S mutant.

These results suggest that the S-H stretching vibration bands observed in the BL and Lumi intermediates primarily arise from Cys87 and Cys299, with Cys211, Cys262, and Cys264 indirectly influencing Cys87 and Cys299 through alterations in hydrogen bonding. Notably, Cys299 is located on TM7 and is only one turn of the α-helix away from Lys296, which forms the Schiff base. According to the AlphaFold2 model structure of MB (Fig. 3 *a*), Cys87 and Cys299 are positioned within hydrogen bonding distance. Interestingly, Cys299 is also situated in a position where it could form a hydrogen bond with the backbone carbonyl group of Lys296 (Fig. S5). Thus, while these residues interact strongly in the initial state, the formation of the Lumi intermediate induces perturbation in TM7, disrupting the triad formed between Cys299, the backbone of Lys296, and Cys87. This disruption likely causes a local movement of TM7, potentially bringing it closer to the counterion Glu113.

Fig. S6 compares the infrared difference spectra of the Lumi intermediates of WT and the C299S mutant. Notably, the 1714 cm^−1^ (+) band, which originates from the protonation of Glu113, is also observed in the C299S mutant, strongly indicating that the C299S mutant forms the Lumi intermediate. However, the characteristic 1661 (−)/1652 (+) cm^−1^ amide-I pair of bands, typically seen in the Lumi intermediate spectrum, is nearly absent in the C299S mutant. Although the α-helix in WT MB linked to the amide-I band changes remains unidentified, current studies with systematic cysteine mutants suggest that these changes are linked to local perturbations in TM7. Therefore, it is reasonable to conclude that, in the C299S mutant, no such perturbation occurs in TM7, resulting in the absence of the amide-I bands.

The proton transfer from the Schiff base to Glu113 in the C299S mutant is believed to result from the mutation-induced perturbation of TM7 in the initial state, which consequently brings the Schiff base and Glu113 into closer proximity. This hypothesis will be explored in more detail in the following section.

### Thermal reisomerization coupled with a proton back-transfer reaction

**Figure 4:**
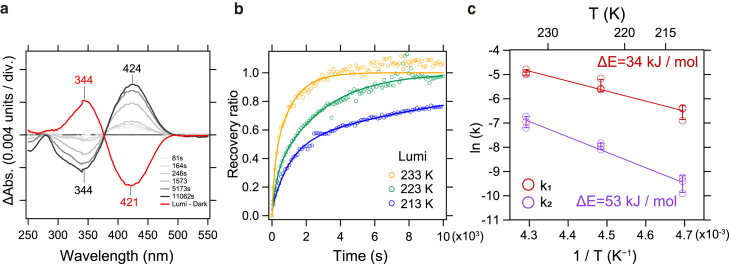
Reversion reaction from MB Lumi intermediate to initial state. (*a*) Time-dependent UV-visible difference spectra measured to monitor the thermal return reaction of Lumi intermediate to the initial state at 223 K. Red line: difference spectra between Lumi intermediate and initial state. Gray-to-black lines: difference spectra representing the process of Lumi intermediate returning to the initial state. (*b*) Temperature dependence of the return reaction from Lumi intermediate to initial state. The *x* axis represents time, and the *y* axis shows the increase in difference absorbance between the λ_max_ of MB (424 nm) and Lumi (344 nm). The return reaction rates at each temperature were calculated by fitting the data with a double exponential function (*solid lines*), and the results are summarized in Table S1. (*c*) Arrhenius plot of the return reaction from Lumi intermediate to initial state within the temperature range of 213–233 K. The natural logarithms of the return reaction rate constants were plotted against the reciprocal of temperature. The slopes of the Arrhenius lines (k_1_: *red line*; k_2_: *purple line*) indicate the activation energies required for the return reaction from the Lumi intermediate to the initial state.

In this study, we observed an unexpected phenomenon where the proton that had transferred from the Schiff base to Glu113 spontaneously returned to the Schiff base. As shown in Fig. 4 *a*, the recovery to the initial state, after light-induced formation of the Lumi intermediate at 223 K (*red solid line*), occurred gradually under dark conditions. After approximately 3 h, the initial state, characterized by absorption at 424 nm, was restored (*black solid line*). Additionally, infrared difference spectrum analysis revealed that, when the recovered initial state—achieved through the spontaneous reprotonation of the Schiff base—was irradiated again, the resulting spectrum (*red line*) perfectly overlapped with the original spectrum (*black line*), as shown in Fig. S7. These findings demonstrate that the reprotonation of Glu113 to the Schiff base is accompanied by retinal isomerization from the all-*trans* form back to the 11-*cis* form.

To calculate the activation energy for the reprotonation reaction from Glu113 to the Schiff base, we performed temperature-dependent measurements of the reaction. As expected, increasing the temperature from 213 K in 10 K increments resulted in an accelerated reaction rate for the return to the initial state (Fig. 4 *b*). The data at each temperature were fitted using a double-exponential function, yielding two rate constants (Table S1). These rate constants at three different temperatures were then plotted on an Arrhenius plot, enabling us to calculate the activation energy for the return to the initial state (Fig. 4 *c*). The activation energies for the fast and slow rate components were found to be 34 and 53 kJ/mol, respectively (Fig. 4 *c*; Table S2), both of which are significantly lower than the photoactivation energy of bovine rhodopsin (>180 kJ/mol) (36,38). This suggests that, from an energetic perspective, the reverse reaction is also thermodynamically feasible.

## Discussion

**Figure 5:**
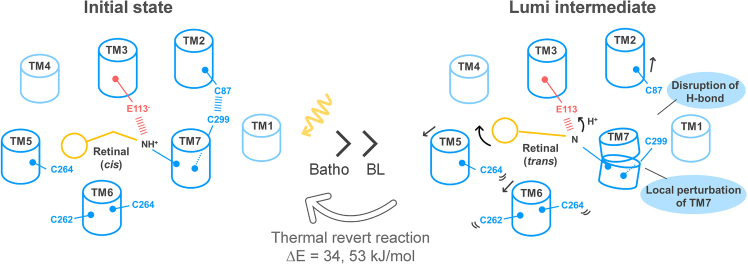
A schematic model illustrating the molecular mechanism behind the structural changes in the MB Lumi intermediate. In the initial state of MB, Glu113 serves as the counterion for the protonated Schiff base. Additionally, Cys87 in TM2 and Cys299 in TM7 form a hydrogen bond. After absorbing light and undergoing thermal relaxation, the Lumi intermediate is formed. This process involves a proton transfer from the Schiff base to Glu113, accompanied by local changes in TM7.These changes result in the disruption of the hydrogen bond between Cys87 and Cys299, causing structural perturbations that propagate to TM5 and TM6. Additionally, the Lumi intermediate undergoes a thermally coupled *trans*-*cis* reisomerization of the retinal, along with a reverse proton transfer from Glu113 to the Schiff base, returning to its initial state. The activation energies for the reaction returning from the Lumi intermediate to the initial state are 34 and 53 kJ/mol, respectively.

In this study, we identified Glu113 as the proton acceptor involved in the deprotonation of the Schiff base, a process that occurs specifically during the formation of the Lumi intermediate in MB. Interestingly, we also discovered that the Lumi intermediate, when trapped at low temperatures, is not stable and eventually reverts to the initial state on a very slow timescale. This transition is accompanied by the reprotonation of the Schiff base by a proton from Glu113, as well as the reisomerization of retinal from the all-*trans* to the 11-*cis* form (Figs. 5 and S8). Additionally, we estimated the activation energy for the return from the Lumi intermediate to the initial state to be between 30 and 60 kJ/mol (Figs. 5 and S8). This is lower than the activation energy for the thermal isomerization of retinal from the 11-*cis* to the all-*trans* form in bovine rhodopsin (∼95 kJ/mol) (36), further supporting the thermodynamic plausibility of our findings.

When MB is exposed to light using a 420 nm interference filter at a temperature of 223 K, both the Lumi and BL intermediate, are observed simultaneously (Fig. 1 *c*). This observation suggests that the BL and Lumi intermediates exist in equilibrium. If this is the case, the BL intermediate, similar to the Lumi intermediate, should spontaneously revert to the initial state. As shown in Fig. S9, the BL intermediate generated at 223 K also undergoes a return to the initial state, indicating that the retinal undergoes thermal reisomerization from the all-*trans* to the 11-*cis* form.

Recent ultrafast transient absorption spectroscopy measurements of HB at room temperature have revealed that the BL intermediate forms within 1 ns, followed by the formation of the Lumi intermediate within 500 ns (28). Notably, the absorption band of the Lumi intermediate lies within the visible spectrum, suggesting that the Schiff base in the Lumi intermediate remains protonated. Given that both HB and MB—the focus of our study—belong to primates and share an exceptionally high amino acid sequence identity of 92% (96% in the vicinity of the retinal binding pocket), this observation may initially appear to contradict our findings. However, considering that the deprotonated Lumi intermediate observed at low temperatures is unstable and exists in equilibrium with the BL intermediate, this apparent discrepancy is understandable. Additionally, transient absorption measurements have captured the intermediate transition rates leading to the active Meta-II intermediate, which forms upon Schiff base deprotonation (28). These measurements indicate that the formation rate of Meta-II in HB is faster than in both bovine rhodopsin and HG, supporting our hypothesis that the Schiff base in MB is inherently more prone to deprotonation.

Moreover, MB and HB are evolutionarily classified as S or SWS1 cone pigments, which are sensitive to violet-ultraviolet light (39). However, in both MB and HB, the initial state absorbs visible light due to the protonation of the Schiff base, indicating an exceptionally high pKa for the Schiff base. In this study, we performed pH titration of MB in its initial state and identified two distinct pKa values: 8.80 (84%) and 10.9 (16%) (Fig. S10). The latter value is likely attributed to the effect of free retinal resulting from protein denaturation. Notably, these pKa values are lower than those observed in rhodopsin (40) as well as red and green pigments (41), further supporting the notion that MB is inherently predisposed to deprotonation and belongs to the S or SWS1 cone pigment family, which typically exists in a deprotonated state in its initial state. It is likely that upon Lumi intermediate formation, structural changes associated with this transition lead to a further decrease in the pKa of the Schiff base, ultimately triggering its deprotonation.

What structural changes, then, facilitate Schiff base deprotonation in the Lumi intermediate? In this study, we used systematic mutagenesis to demonstrate that the physical proximity between the Schiff base and Glu113 is the key structural factor enabling the proton transfer from the Schiff base to Glu113 (and the reverse reaction) during Lumi intermediate formation in MB. Initially, it was anticipated that local perturbations in TM3 during Lumi intermediate formation would bring Glu113 closer to the Schiff base. However, contrary to this expectation, based on mutagenesis and FTIR data (Fig. 3 *c*), we found that S-H vibrations in BL and Lumi intermediates originate from Cys87 and Cys299. According to our AlphaFold2-based model (Fig. 3 *a*), Cys299 is positioned to interact with the backbone carbonyl of Lys296. Lumi formation appears to perturb this triad (Cys87-Cys299-Lys296), likely causing local movement of TM7 toward Glu113. Previous FTIR spectroscopy studies in bovine rhodopsin (23) and MG (32) have shown that during Lumi intermediate formation, the hydrogen-bonding strength of the Schiff base weakens. Furthermore, structural comparisons between the initial state and the Lumi intermediate in bovine rhodopsin reveal that following retinal isomerization, the outward displacement of TM3 causes Glu113 to move away from the Schiff base, thereby weakening the hydrogen bond (11). Taken together, these findings suggest that the structural perturbations near TM7 during the photoreaction process in MB differ from those observed in other visual pigments, indicating that the structural changes, or dynamics, accompanying Lumi intermediate formation in MB are uniquely distinct.

An example of retinal movement driven by perturbation of the Schiff base-forming lysine residue has been experimentally observed in C1C2 channelrhodopsin, a microbial rhodopsin. Time-resolved x-ray crystallography of C1C2 channelrhodopsin revealed that, during the early stage of the photoreaction (∼1 ms), the lysine residue shifts inward into the membrane after retinal isomerization, causing TM3 to move outward (42). This initial displacement triggers a cascade of structural changes in adjacent TM2 and TM6, ultimately leading to channel opening. These structural changes have been further supported by time-resolved FTIR spectroscopy and QM/MM simulations. Notably, the amide-I band observed at 1 ms in the time-resolved FTIR spectrum (1664 (−)/1648 (+) cm^−1^) closely resembles that of the Lumi intermediate in MB, which exhibits a band at 1661 (−)/1652 (+) cm^−1^. During Lumi intermediate formation in MB, the perturbation of the Schiff base is primarily transmitted through TM7, influencing adjacent TM2, TM5, and TM6, as demonstrated by measurements using systematic cysteine mutants.

To further support the MB-specific perturbation of TM7 near the Schiff base, we focused on the S-H stretching vibration bands of cysteine residues, which were prominently observed during Lumi intermediate formation (Fig. 3 *c*, *black line*). Through a systematic mutagenesis approach targeting specific cysteine residues, we successfully identified structural perturbations of TM7 in the Lumi intermediate. The S-H stretching vibration band of cysteine residues can be observed independently from other functional group vibration modes, making it a highly effective probe for detecting local structural changes in proteins. In fact, previous studies on rhodopsin have demonstrated that cysteine residue mutations have enabled the utilization of the S-H stretching vibration band as a probe to track α-helix structural changes during the transition between photoreaction intermediates (43). Applying a similar approach to cone pigments in future studies is expected to further enhance our understanding of the structural dynamics in these proteins.

On the other hand, the structural factors regulating the pKa of the Schiff base in the initial state of MB remain unclear. Previous studies have suggested that the amino acid at position 86 plays a role in modulating the Schiff base’s pKa (44). Therefore, further research focusing on this site in MB is anticipated to provide deeper insights into this mechanism.

Additionally, the amino acid at position 86 plays a crucial role in the activation of visual pigments. Recent time-resolved x-ray crystallography studies of bovine rhodopsin have revealed that a hydrogen-bonding network, mediated by water, forms between the counterion Glu113 and Met86 (45). This network undergoes reorganization during the formation of the Batho intermediate. In our recent study, we found that the glutamic acid at position 86 in MG remains protonated throughout the transition from the initial state to the Lumi intermediate (32). Moreover, we observed changes in the strength of the hydrogen bonds during the transitions between photoreaction intermediates.

These findings suggest that in both MG and bovine rhodopsin, retinal isomerization during the early stages of the photoreaction propagates through the amino acid at position 86 of TM2. Furthermore, changes in the hydrogen bonding of the S-H group of Cys87, located just above position 86, were observed in MB. This observation implies that MB may have an activation pathway where retinal isomerization is transmitted through TM2. However, the dynamics of structural changes mediated by TM2 during the early stages of the photoreaction likely differ between visual pigments. Specifically, our measurements indicate that, in MB, a hydrogen-bonding network forms in the initial state between Cys87 in TM2, Cys299 in TM7, and Lys296 in TM7. This network undergoes rearrangement during the transition to the Lumi intermediate.

Interestingly, previous crystallographic studies comparing the initial, Lumi, and active intermediate states of bovine rhodopsin (in complex with the C-terminal peptide of the G-protein) revealed that the 3.10-helix structure formed between Lys296 and Ala299 of TM7 in the initial state remains largely unchanged in the Lumi intermediate but transitions to a more α-helix-like structure upon activation (9). This suggests that the structural transition observed in bovine rhodopsin may already be occurring at the Lumi intermediate stage in MB. Moreover, given that Cys87 and Cys299 are highly conserved within the S or SWS1 cone pigment group, these findings imply that MB may share photoreaction dynamics with other S or SWS1 cone pigments, highlighting common features in their activation mechanisms.

## Conclusion

A comprehensive analysis of the photoreaction processes in visual pigments offers valuable insights into the molecular mechanisms of phototransduction, specifically how absorbed light energy is converted into conformational changes within the protein, ultimately leading to receptor activation. In this study, we identified unique phenomena during the formation of the Lumi intermediate in MB, including proton transfer from the retinal Schiff base to Glu113 and localized perturbations in TM7. These observations, which have not been reported in other animal rhodopsins, suggest that these characteristics are specific to MB and may be closely linked to the molecular evolution of animal rhodopsins.

Future studies examining the late intermediates of MB and the spectral properties of UV-sensitive visual pigments are poised to offer deeper insights into the molecular basis of phototransduction and enhance our understanding of the evolutionary processes of animal rhodopsins. Indeed, in rhodopsin, the E113D mutation does not appear to significantly impair activation (46), suggesting that the functional role of Glu113 in phototransduction may differ between rhodopsin and cone pigments. Moreover, since visual pigments are part of the G-protein-coupled receptor (GPCR) family, which plays a key role in regulating a wide range of physiological processes in humans, understanding the activation mechanisms of visual pigments will not only contribute to unraveling the functional mechanisms of other GPCRs but also aid in advancing GPCR-targeted drug discovery.

## Acknowledgments

We thank Dr. Gulati S (Thermo Inc.) for valuable comments on this manuscript. We also express our deep gratitude to Ms. Shino Inukai for providing the schematic model illustration. This research was supported in part by grants from JST, the 10.13039/501100002241Japan Science and Technology Agency to K.K. (JPMJPR19G4), and from the 10.13039/501100001700Japanese Ministry of Education, Culture, Sports, Science, and Technology to H.K. (21H04969), Grant-in-Aid for Scientific Research on Innovative Areas “Non-equilibrium-state molecular movies and their applications (Molecular Movies)” from 10.13039/501100001700MEXT, Japan, to K.K. (20H05440) and Y.M. (JP24KJ1312). This study was also financially supported by Research Units for Exploring Future Horizons and Future Development Research Funding Program, 10.13039/501100013226Kyoto University Research Coordination Alliance.

## Author contributions

K.K. and H.K. conceived and designed the experiments. Y.M. prepared samples and performed all experiments with the help of K.K. Y.M. analyzed the data with the help of K.K. K.K., Y.M., and H.K. wrote the manuscript. K.K., Y.M., H.I., and H.K. coordinated and oversaw the research project. All authors discussed the results and commented on the manuscript.

## Declaration of interests

The authors declare that they have no conflicts of interest with the contents of this article.

## Supporting material

Document S1. Figures S1–S10 and Tables S1 and S2

Document S2. Article plus supporting material

## References

[1] Wald G. (1968). The Molecular Basis of Visual Excitation. Nature.

[2] Palczewski K. (2006). G Protein–Coupled Receptor Rhodopsin. Annu. Rev. Biochem..

[3] Ernst O.P., Lodowski D.T., Kandori H. (2014). Microbial and Animal Rhodopsins: Structures, Functions, and Molecular Mechanisms. Chem. Rev..

[4] Shichida Y., Imai H. (1998). Visual pigment: G-protein-coupled receptor for light signals. Cell. Mol. Life Sci..

[5] Oprian D.D., Asenjo A.B., Pelletier S.L. (1991). Design, chemical synthesis, and expression of genes for the three human color vision pigments. Biochemistry.

[6] Ebrey T., Koutalos Y. (2001). Vertebrate Photoreceptors. Prog. Retin. Eye Res..

[7] Palczewski K., Kumasaka T., Miyano M. (2000). Crystal Structure of Rhodopsin: A G Protein-Coupled Receptor. Science.

[8] Okada T., Sugihara M., Buss V. (2004). The Retinal Conformation and its Environment in Rhodopsin in Light of a New 2.2 Å Crystal Structure. J. Mol. Biol..

[9] Li J., Edwards P.C., Schertler G.F.X. (2004). Structure of Bovine Rhodopsin in a Trigonal Crystal Form. J. Mol. Biol..

[10] Nakamichi H., Okada T. (2006). Crystallographic Analysis of Primary Visual Photochemistry. Angew. Chem. Int. Ed..

[11] Nakamichi H., Okada T. (2006). Local peptide movement in the photoreaction intermediate of rhodopsin. Proc. Natl. Acad. Sci. USA.

[12] Choe H.-W., Kim Y.J., Ernst O.P. (2011). Crystal structure of metarhodopsin II. Nature.

[13] Deupi X., Edwards P., Standfuss J. (2012). Stabilized G protein binding site in the structure of constitutively active metarhodopsin-II. Proc. Natl. Acad. Sci. USA.

[14] Tsai C.-J., Pamula F., Schertler G.F.X. (2018). Crystal structure of rhodopsin in complex with a mini-Go sheds light on the principles of G protein selectivity. Sci. Adv..

[15] Ruprecht J.J., Mielke T., Schertler G.F.X. (2004). Electron crystallography reveals the structure of metarhodopsin I. EMBO J..

[16] Kang Y., Kuybeda O., Xu H.E. (2018). Cryo-EM structure of human rhodopsin bound to an inhibitory G protein. Nature.

[17] Tsai C.-J., Marino J., Schertler G. (2019). Cryo-EM structure of the rhodopsin-Gαi-βγ complex reveals binding of the rhodopsin C-terminal tail to the gβ subunit. Elife.

[18] Gao Y., Hu H., Skiniotis G. (2019). Structures of the Rhodopsin-Transducin Complex: Insights into G-Protein Activation. Mol. Cell.

[19] Bagley K.A., Balogh-Nair V., Vittitow J. (1985). Fourier-transform infrared difference spectroscopy of rhodopsin and its photoproducts at low temperature. Biochemistry.

[20] Ganter U.M., Gärtner W., Siebert F. (1988). Rhodopsin-lumirhodopsin phototransition of bovine rhodopsin investigated by Fourier transform infrared difference spectroscopy. Biochemistry.

[21] Kandori H., Maeda A. (1995). FTIR Spectroscopy Reveals Microscopic Structural Changes of the Protein around the Rhodopsin Chromophore upon Photoisomerization. Biochemistry.

[22] Bartl F., Ritter E., Hofmann K.P. (2000). FTIR spectroscopy of complexes formed between metarhodopsin II and C-terminal peptides from the G-protein α- and γ-subunits. FEBS Lett..

[23] Furutani Y., Shichida Y., Kandori H. (2003). Structural Changes of Water Molecules during the Photoactivation Processes in Bovine Rhodopsin. Biochemistry.

[24] Zaitseva E., Brown M.F., Vogel R. (2010). Sequential Rearrangement of Interhelical Networks Upon Rhodopsin Activation in Membranes: The Meta II a Conformational Substate. J. Am. Chem. Soc..

[25] Ehrenberg D., Varma N., Lesca E. (2019). The Two-Photon Reversible Reaction of the Bistable Jumping Spider Rhodopsin-1. Biophys. J..

[26] Imai H., Terakita A., Shichida Y. (1997). Photochemical and Biochemical Properties of Chicken Blue-Sensitive Cone Visual Pigment. Biochemistry.

[27] Dhankhar D., Salom D., Rentzepis P.M. (2023). Ultrafast spectra and kinetics of human green-cone visual pigment at room temperature. Proc. Natl. Acad. Sci. USA.

[28] Krishnamoorthi A., Salom D., Rentzepis P.M. (2024). Ultrafast transient absorption spectra and kinetics of human blue cone visual pigment at room temperature. Proc. Natl. Acad. Sci. USA.

[29] Katayama K., Furutani Y., Kandori H. (2010). An FTIR Study of Monkey Green- and Red-Sensitive Visual Pigments. Angew. Chem. Int. Ed. Engl..

[30] Katayama K., Nonaka Y., Kandori H. (2017). Spectral Tuning Mechanism of Primate Blue-sensitive Visual Pigment Elucidated by FTIR Spectroscopy. Sci. Rep..

[31] Hanai S., Katayama K., Kandori H. (2021). Light-induced difference FTIR spectroscopy of primate blue-sensitive visual pigment at 163 K. Biophys. Physicobiol..

[32] Sasaki T., Katayama K., Kandori H. (2024). Glu1022.53-Mediated Early Conformational Changes in the Process of Light-Induced Green Cone Pigment Activation. Biochemistry.

[33] Mizuno Y., Katayama K., Kandori H. (2022). Early Proton Transfer Reaction in a Primate Blue-Sensitive Visual Pigment. Biochemistry.

[34] Sakmar T.P., Franke R.R., Khorana H.G. (1991). The role of the retinylidene Schiff base counterion in rhodopsin in determining wavelength absorbance and Schiff base pKa. Proc. Natl. Acad. Sci. USA.

[35] Hirano T., Fujioka N., Shichida Y. (2006). Assignment of the Vibrational Modes of the Chromophores of Iodopsin and Bathoiodopsin: Low-Temperature Fourier Transform Infrared Spectroscopy of 13C- and 2H-Labeled Iodopsins. Biochemistry.

[36] Lórenz-Fonfría V.A., Furutani Y., Kandori H. (2010). Protein Fluctuations as the Possible Origin of the Thermal Activation of Rod Photoreceptors in the Dark. J. Am. Chem. Soc..

[37] Nagata T., Oura T., Shichida Y. (2002). Isomer-Specific Interaction of the Retinal Chromophore with Threonine-118 in Rhodopsin. J. Phys. Chem. A.

[38] Okada T., Ernst O.P., Hofmann K.P. (2001). Activation of rhodopsin: new insights from structural and biochemical studies. Trends Biochem. Sci..

[39] Okano T., Kojima D., Yoshizawa T. (1992). Primary structures of chicken cone visual pigments: vertebrate rhodopsins have evolved out of cone visual pigments. Proc. Natl. Acad. Sci. USA.

[40] Steinberg G., Ottolenghi M., Sheves M. (1993). pKa of the protonated Schiff base of bovine rhodopsin. A study with artificial pigments. Biophys. J..

[41] Kochendoerfer G.G., Wang Z., Mathies R.A. (1997). Resonance Raman Examination of the Wavelength Regulation Mechanism in Human Visual Pigments. Biochemistry.

[42] Oda K., Nomura T., Nureki O. (2021). Time-resolved serial femtosecond crystallography reveals early structural changes in channelrhodopsin. Elife.

[43] Yamazaki Y., Nagata T., Imamoto Y. (2014). Intramolecular Interactions That Induce Helical Rearrangement upon Rhodopsin Activation: LIGHT-INDUCED STRUCTURAL CHANGES IN METARHODOPSIN IIa PROBED BY CYSTEINE S-H STRETCHING VIBRATIONS. J. Biol. Chem..

[44] Sekharan S., Mooney V.L., Batista V.S. (2013). Spectral Tuning of Ultraviolet Cone Pigments: An Interhelical Lock Mechanism. J. Am. Chem. Soc..

[45] Gruhl T., Weinert T., Panneels V. (2023). Ultrafast structural changes direct the first molecular events of vision. Nature.

[46] Jäger S., Lewis J.W., Kliger D.S. (1997). Chromophore structural changes in rhodopsin from nanoseconds to microseconds following pigment photolysis. Proc. Natl. Acad. Sci. USA.

