# Supplementary material for: Deprotonation of retinal Schiff base and structural dynamics in the early photoreaction of primate blue cone visual pigment
Source: Biophys J. 2025 May 7;124(12):2070–81. doi: 10.1016/j.bpj.2025.05.004 (PMC12256820; doi:10.1016/j.bpj.2025.05.004)
Supplement: Document S1. Figures S1–S10 and Tables S1 and S2 [file mmc1.pdf]

Biophysical Journal, Volume *124*

Supplemental information

Deprotonation of retinal Schiff base and structural dynamics in the early photoreaction of primate blue cone visual pigment

Yosuke Mizuno, Kota Katayama, Hiroo Imai, and Hideki Kandori

Supporting Information

Deprotonation of Retinal Schiff Base and Structural Dynamics in the Early Photoreaction of Primate Blue Cone Visual Pigment

Yosuke Mizuno[1], Kota Katayama[1,2*], Hiroo Imai[3], and Hideki Kandori[1,2*]

[1]Department of Life Science and Applied Chemistry, Nagoya Institute of Technology, Showa-ku, Nagoya 466-8555, Japan

[2]OptoBioTechnology Research Center, Nagoya Institute of Technology, Showa-ku, Nagoya 466-8555, Japan

[3]Center for the Evolutionary Origins of Human Behavior, Kyoto University, Inuyama 484-8506, Japan

[*]To whom correspondence should be addressed. E-mail: katayama.kota@nitech.ac.jp (K.K.), kandori@nitech.ac.jp (H.K.)

Contents

Supporting Figures S1-S10.

Table S1,2

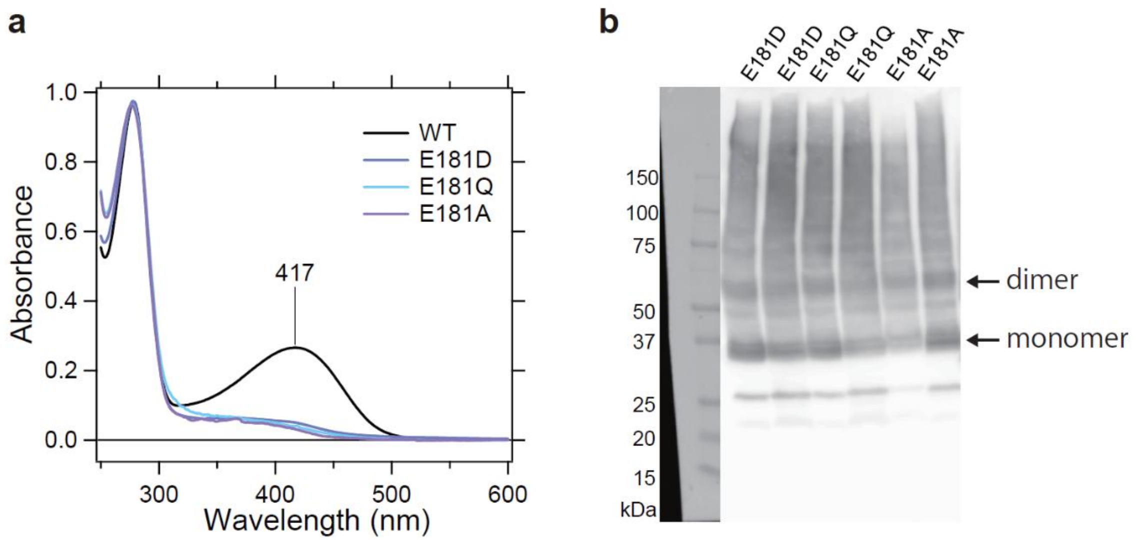

Figure S1. (*a*) UV-visible absorption spectra of WT and various E181 mutants after purification. (*b*) Western blot analysis of various E181 mutants.

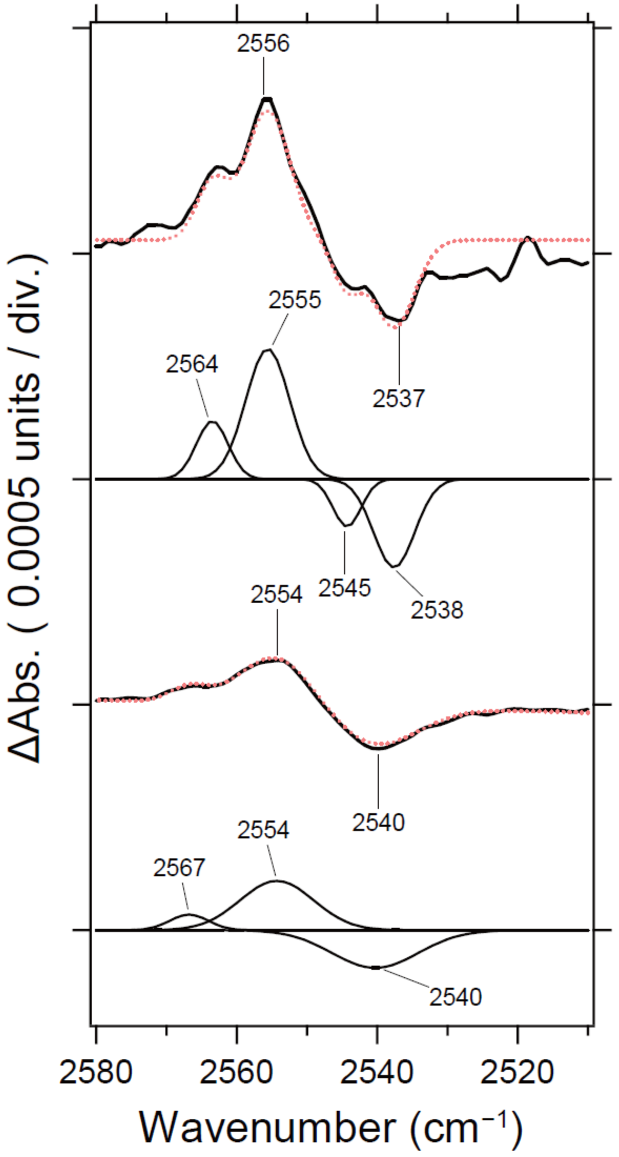

Figure S2. Deconvolution analysis of the S-H stretching vibration band of cysteine 1st row: Measured S-H stretching vibration band of cysteine in the Lumi intermediate (black solid line) and its Gaussian fitting curve (red dashed line). 2nd row: Deconvoluted Gaussian fitting curves of the red dashed line in the 1st row, with two components on the positive side and two on the negative side. 3rd row: Measured S-H stretching vibration band of cysteine in the BL intermediate (black solid line) and its Gaussian fitting curve (red dashed line). 4th row: Deconvoluted Gaussian fitting curves of the red dashed line in the 3rd row, with two components on the positive side and one on the negative side.

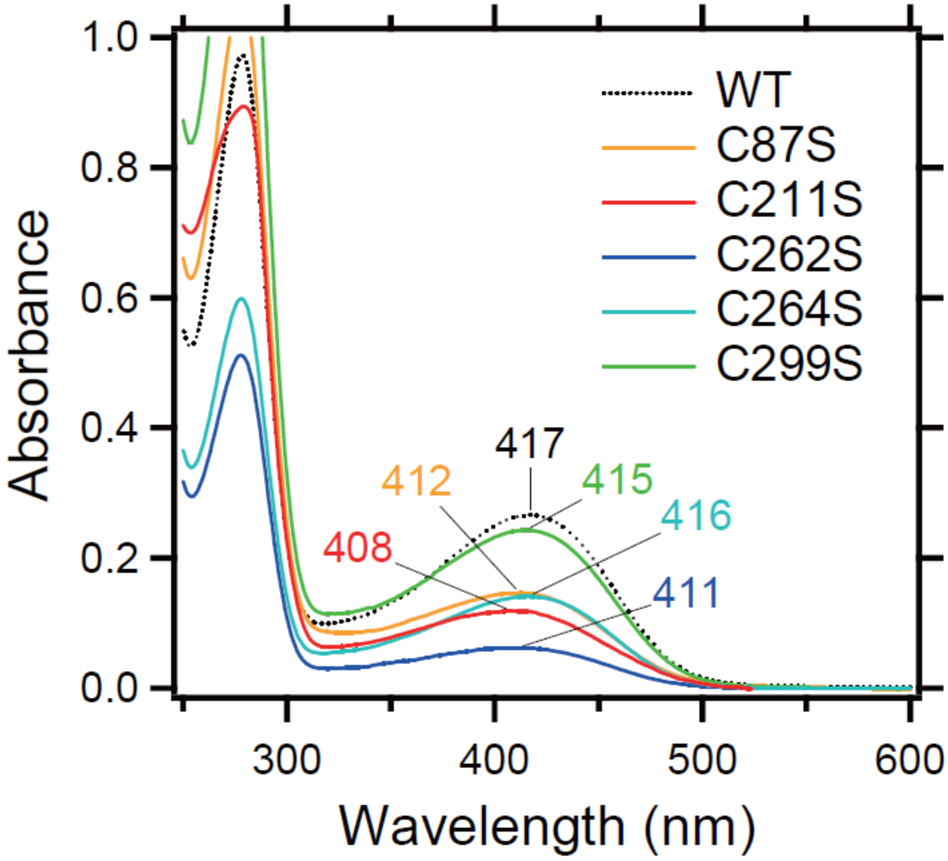

Figure S3. UV-visible absorption spectra of WT and various cysteine mutants after purification.

a

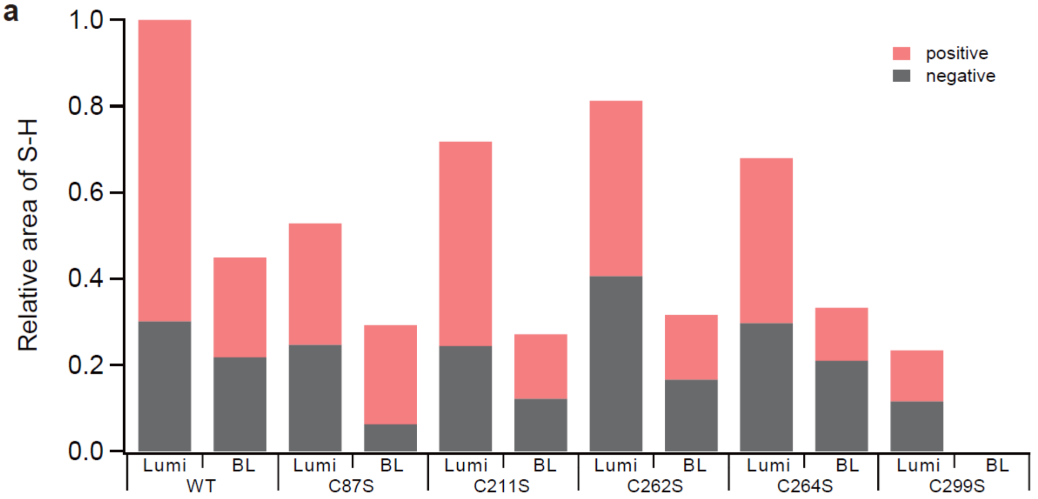

b

	MB WT		C87S		C211S		C262S		C264S		C299S	
	Lumi	BL	Lumi	BL	Lumi	BL	Lumi	BL	Lumi	BL	Lumi	BL
positive	0.70	0.23	0.28	0.23	0.47	0.15	0.41	0.15	0.38	0.12	0.12	-
negative	0.30	0.22	0.25	0.06	0.24	0.12	0.41	0.17	0.30	0.21	0.12	-
p/n	2.3	1.1	1.1	3.7	1.9	1.2	1.0	0.91	1.3	0.59	1.0	-

Figure S4. Comparison of the integrated areas of S-H stretching vibration bands in WT and various cysteine mutants. (*a*, *b*) Comparison of the negative (Initial state) and positive (BL/Lumi) S-H stretching vibration bands observed in WT and each mutant in Figure 3c.

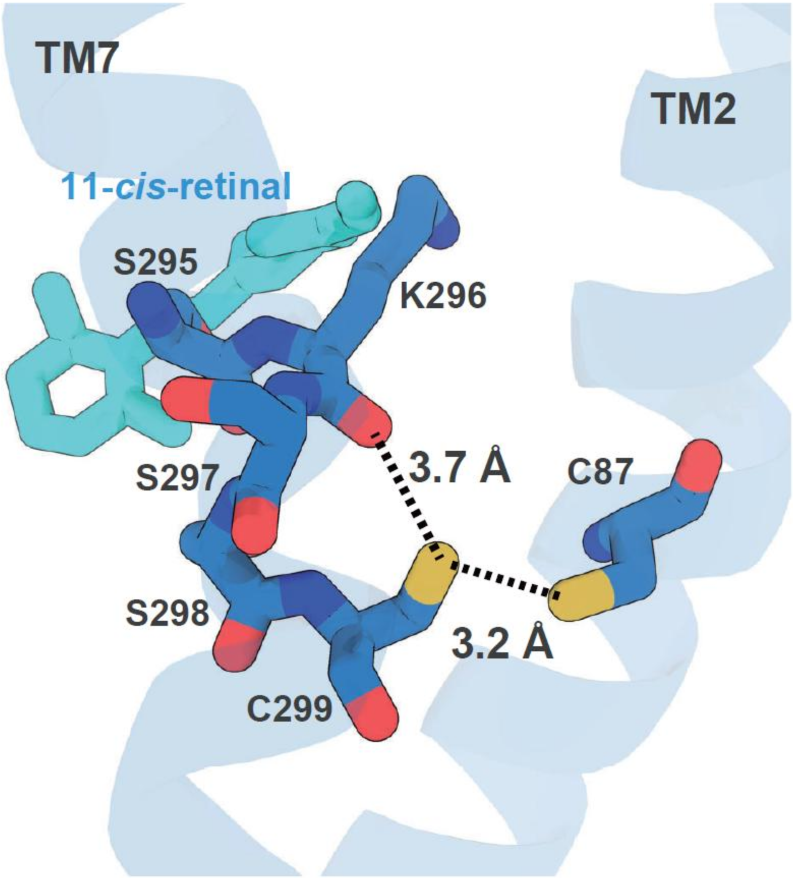

Figure S5. Structure of the MB model generated using AlphaFold2, viewed from the TM7 side. The 11-*cis*-retinal was positioned based on the structure of bovine rhodopsin (PDB ID: 1U19).

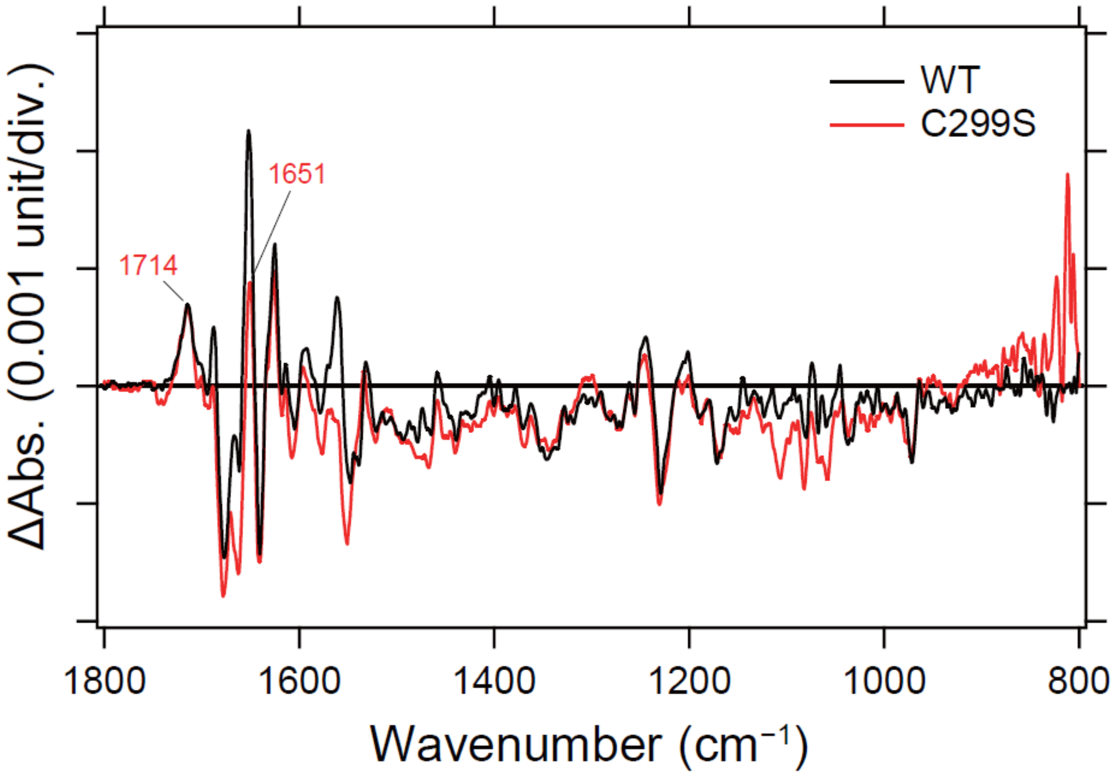

Figure S6. Comparison of the light-induced FTIR difference spectra of the Lumi intermediate for WT (black line) and the C299S mutant (red line). The 1714 cm^{-1} and 1651 cm^{-1} bands correspond to the protonated C=O stretching vibration of Glu113 and the amide-I band, respectively, both of which are specific to the MB-Lumi intermediate.

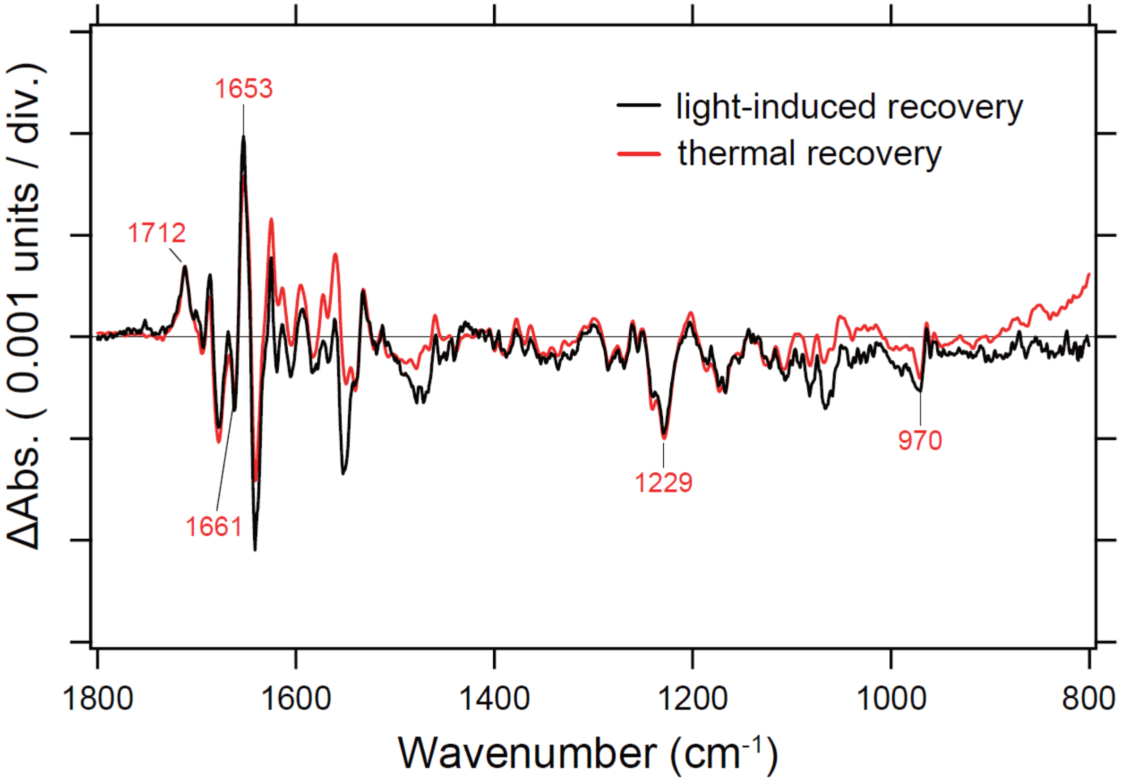

Figure S7. Comparison of the light-induced FTIR difference spectra of the Lumi intermediate formed upon the second illumination, following either light-induced recovery (black line) or thermal recovery (red line) of MB.

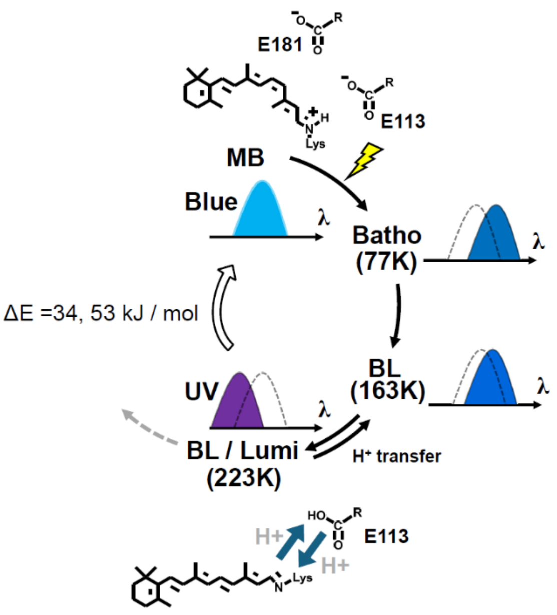

Figure S8. Photo reaction scheme of MB up to Lumi.

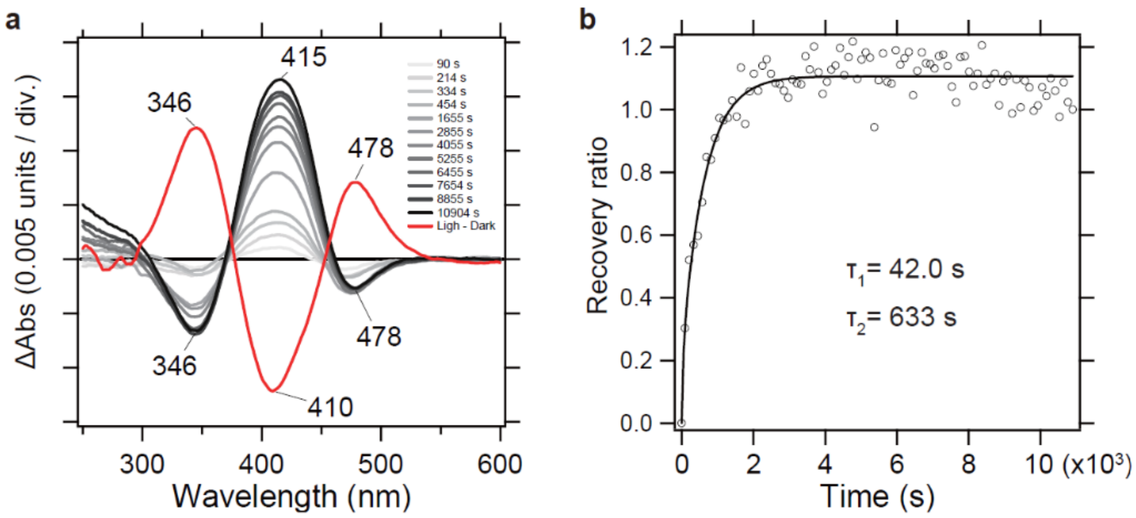

Figure S9. Reversion reaction from MB BL intermediate to initial state. (*a*) Time-dependent UV-visible difference spectra measured to monitor the thermal return reaction of BL intermediate to the initial state at 223 K. Red line: Difference spectra between BL/Lumi intermediates and initial state. Gray-to-black lines: Difference spectra representing the process of BL/Lumi intermediates returning to the initial state. (*b*) Return reaction kinetics from BL/Lumi intermediates to initial state. The X-axis represents time and the Y-axis shows the increase in difference absorbance between the λ_{max} of MB (415 nm) and BL (478 nm). The return reaction rate was calculated by fitting the data with a double exponential function (solid line).

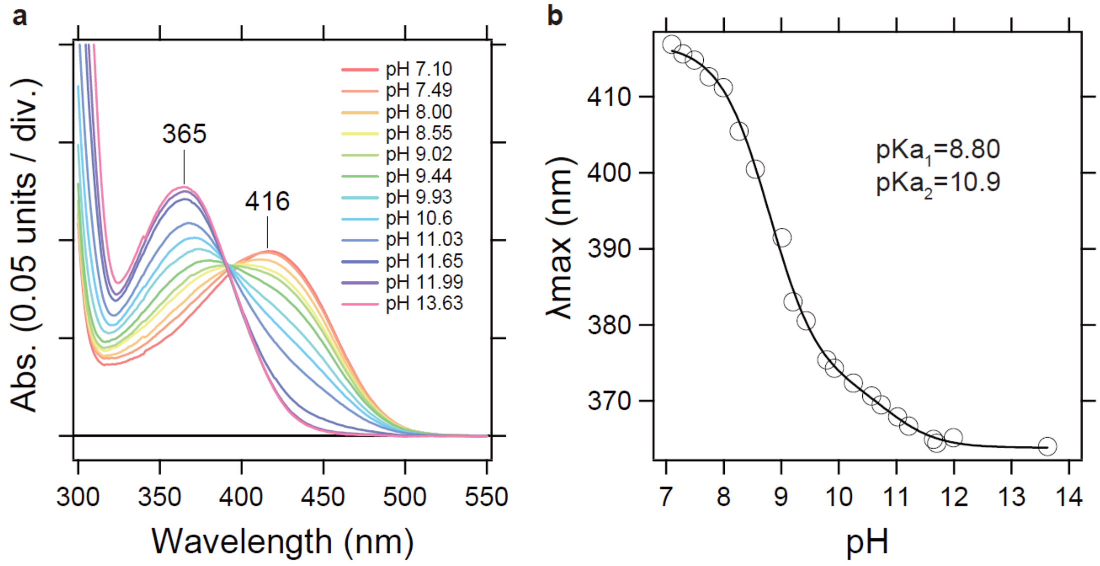

Figure S10. pH-dependent absorption changes in MB. (*a*) Absorption spectra of MB at pH 7.10 to 13.63. (*b*) pH-dependent changes of λ_{max}. The titration curve was analyzed using the Henderson-Hasselbalch equation assuming double pK_a values (solid line).

Table S1. The return reaction rates at each temperature.

	213 K		223 K		233 K	
	k_1 (s^{-1})	k_2 (s^{-1})	k_1 (s^{-1})	k_2 (s^{-1})	k_1 (s^{-1})	k_2 (s^{-1})
1	1.00×10^{-3}	8.24×10^{-5}	3.60×10^{-3}	3.06×10^{-4}	6.71×10^{-3}	7.47×10^{-4}
2	1.68×10^{-3}	9.63×10^{-5}	3.79×10^{-3}	3.54×10^{-4}	8.31×10^{-3}	11.86×10^{-4}
3	1.65×10^{-3}	4.99×10^{-5}	5.70×10^{-3}	3.94×10^{-4}	7.08×10^{-3}	9.54×10^{-4}

Table S2. Values of the Arrhenius analysis and activation energy required for the return reaction.

	a	b	R^2	ΔE (kJ / mol)
k_1	12.8 ± 2.3	$-4.12 \times 10^3 \pm 0.51 \times 10^3$	-0.950	34
k_2	20.4 ± 2.4	$-6.36 \times 10^3 \pm 0.52 \times 10^3$	-0.977	53
